# Racial and Ethnic Differences in Factors Associated With Delayed or Missed Pediatric Preventive Care in the US Due to the COVID-19 Pandemic

**DOI:** 10.1001/jamanetworkopen.2023.22588

**Published:** 2023-07-10

**Authors:** Maya Tabet, Russell S. Kirby, Pamela Xaverius

**Affiliations:** 1 College of Global Population Health, University of Health Sciences and Pharmacy in St Louis, St Louis, Missouri; 2 Chiles Center, College of Public Health, University of South Florida, Tampa; 3 Office of Research and Scholarly Activity, University of Health Sciences and Pharmacy in St Louis, St Louis, Missouri

## Abstract

**Importance:**

The COVID-19 pandemic has disrupted medical care use. Yet information on how the pandemic has affected pediatric preventive care use in the US is lacking.

**Objective:**

To examine the prevalence of and risk and protective factors for delayed or missed pediatric preventive care in the US due to the COVID-19 pandemic, further stratified by race and ethnicity to assess associations by groups.

**Design, Setting, and Participants:**

This cross-sectional study used data from the 2021 National Survey of Children’s Health (NSCH) collected between June 25, 2021, and January 14, 2022. Weighted data from the NSCH survey are representative of the population of noninstitutionalized children aged 0 to 17 years in the US. For this study, race and ethnicity were reported as American Indian or Alaska Native, Asian or Pacific Islander, Hispanic, non-Hispanic Black, non-Hispanic White, or multiracial (≥2 races). Data analysis was performed on February 21, 2023.

**Exposures:**

The Andersen behavioral model of health services use was used to assess predisposing factors, enabling factors, and need factors.

**Main Outcomes and Measures:**

The main outcome was delayed or missed pediatric preventive care due to the COVID-19 pandemic. Bivariate and multivariable Poisson regression analyses were performed using multiple imputation with chained equations.

**Results:**

Of the 50 892 NSCH respondents, 48.9% were female and 51.1% were male; their mean (SD) age was 8.5 (5.3) years. With regard to race and ethnicity, 0.4% were American Indian or Alaska Native, 4.7% were Asian or Pacific Islander, 13.3% were Black, 25.8% were Hispanic, 50.1% were White, and 5.8% were multiracial. More than one-fourth of children (27.6%) delayed or missed preventive care. In multivariable Poisson regression using multiple imputation, Asian or Pacific Islander children (prevalence ratio [PR], 1.16 [95% CI, 1.02-1.32]), Hispanic children (PR, 1.19 [95% CI, 1.09-1.31]), and multiracial children (PR, 1.23 [95% CI, 1.11-1.37]) were more likely to have delayed or missed preventive care compared with non-Hispanic White children. Among non-Hispanic Black children, risk and protective factors included age 6 to 8 years (vs 0-2 years [PR, 1.90 (95% CI, 1.23-2.92)]) and difficulty covering basic needs somewhat or very often (vs never or rarely [PR, 1.68 (95% CI, 1.35-2.09)]). Among multiracial children, risk and protective factors included age 9 to 11 years (vs 0-2 years [PR, 1.73 (95% CI, 1.16-2.57)]), lower household income (<100% vs ≥400% federal poverty level: 0.52 [0.35-0.79]), and 2 or more vs 0 health conditions (PR, 1.54 [95% CI, 1.14-2.08]). Among non-Hispanic White children, risk and protective factors included older age (9-11 years vs 0-2 years [PR, 2.05 (95% CI, 1.78-2.37)]), 4 or more children vs 1 child in the household (PR, 1.22 [95% CI, 1.07-1.39]), fair or poor vs excellent or very good caregiver health (PR, 1.32 [95% CI, 1.18-1.47]), difficulty covering basic needs somewhat or very often (vs never or rarely [PR, 1.36 (95% CI, 1.22-1.52)]), good vs excellent or very good perceived child health (PR, 1.19 [95% CI, 1.06-1.34]), and 2 or more vs 0 health conditions (PR, 1.25 [95% CI, 1.12-1.38]).

**Conclusions and Relevance:**

In this study, the prevalence of and risk factors for delayed or missed pediatric preventive care varied by race and ethnicity. These findings may guide targeted interventions to enhance timely pediatric preventive care among different racial and ethnic groups.

## Key Points

**Question:**

What risk and protective factors are associated with delayed or missed pediatric preventive care in the US due to the COVID-19 pandemic by racial and ethnic group?

**Findings:**

In this cross-sectional study using data from 50 892 respondents to the 2021 National Survey of Children’s Health, 27.6% of children had delayed or missed preventive care due to the COVID-19 pandemic. Predisposing, enabling, and need factors associated with delayed or missed preventive care differed by racial and ethnic group.

**Meaning:**

These findings highlight factors that may guide the design of targeted interventions to enhance timely pediatric preventive care among different racial and ethnic groups.

## Teaser

This cross-sectional study uses data from the 2021 National Survey of Children’s Health to assess the prevalence of and risk and protective factors for delayed or missed preventive care in the US due to the COVID-19 pandemic, stratified by race and ethnicity.

## Introduction

During the COVID-19 pandemic, lockdown restrictions, social distancing, limitations in access to resources, and fear of COVID-19 exposure in health care settings led to disruptions in health care access and use.^1,2^ Although data among children remain limited, available evidence highlights delayed or missed pediatric preventive care,^3,4,5,6^ with serious implications. Indeed, studies have highlighted reductions in routine childhood immunizations during times of quarantine,^7,8^ increasing the potential for a resurgence in preventable diseases and associated mortality.^8^ Studies have also described delayed diagnosis and treatment of a variety of conditions, thereby increasing the risk of morbidity, mortality, disability, preventable intensive care admissions, and extensive health care expenditures.^9,10,11,12,13,14,15,16,17^

Studies examining the prevalence of delayed or missed pediatric preventive care during the COVID-19 pandemic in a nationwide sample of US children are scarce, and only a few have explored potential determinants.^4,5^ Such studies did not examine potential risk factors such as child age, main caregiver physical or mental health, home ownership, having a usual source of pediatric preventive care, having a personal doctor or nurse, perceived child health, and evaluated child health,^4,5^ which may have resulted in residual confounding, because such factors may affect health care access and use.^18,19^ Further, previous studies^4,5^ did not examine associations by child racial and ethnic group, which may have precluded the identification of race and ethnicity–specific risk and protective factors for delayed or missed pediatric preventive care.

Improved understanding of the extent to which children in the US are receiving the care they need may help identify children who would benefit from programs and services to promote timely pediatric preventive care visits. The primary objective of this study was to examine the prevalence of and risk and protective factors for delayed or missed pediatric preventive care due to the COVID-19 pandemic in a nationwide sample of US children (aged 0-17 years). Because different racial and ethnic groups may experience different barriers to health care services use^20^ and given the disproportionate repercussions of the pandemic on different racial and ethnic groups,^21^ our secondary objective was to examine associations by race and ethnicity.

## Methods

### Data Source and Study Population

This cross-sectional study used data from the 2021 National Survey of Children’s Health (NSCH), an annual nationally representative survey of children aged 0 to 17 years.^22^ The NSCH is funded by the Maternal and Child Health Bureau of the US Department of Health and Human Services Health Resources and Services Administration and conducted by the US Census Bureau. The survey provides data on sociodemographic characteristics, health and well-being, and health care access and quality.^23^ In 2021, questions were added to evaluate the association between the COVID-19 pandemic and health care use.^24^ Households across the US were randomly selected and contacted by mail to identify those with 1 or more children aged 0 to 17 years. Caregivers completed the questionnaire for 1 randomly selected child, online or on paper between June 25, 2021, and January 14, 2022.^25^ Because all information was deidentified, the University of Health Sciences and Pharmacy in St Louis Institutional Review Board classified this study as exempt from review and informed consent was waived. The study followed the Strengthening the Reporting of Observational Studies in Epidemiology (STROBE) reporting guideline.

### Definition of Variables

We examined predisposing, enabling, and need factors, as guided by the Andersen behavioral model of health services use.^26,27^

#### Predisposing Factors

Predisposing factors included the following: child age (0-2, 3-5, 6-8, 9-11, 12-14, or 15-17 years), child sex (male or female), caregiver highest level of education (less than high school or high school or more), family structure (2-parent, single-mother, single-father, or grandparent or other household type), total number of children in the household (1, 2, 3, or ≥4), total number of children with special health care needs in the household (0, 1, or ≥2), and a composite measure of main caregiver physical and mental health (excellent or very good [both self-reported as excellent or very good], good [at least 1 indicator self-reported as good and the other self-reported as good, very good, or excellent], and fair or poor [either mental health or physical health self-reported as fair or poor]). Child racial or ethnic group was included as a predisposing variable in the overall analysis and as a stratifying variable in the stratified analysis. This variable was constructed from race (American Indian or Alaska Native alone, Asian alone, Black or African American alone, Native Hawaiian and other Pacific Islander alone, White alone, or ≥2 races) and ethnicity (Hispanic or Latino origin or not) reported by the parent or caregiver. For this study, racial and ethnic groups are reported as follows: American Indian or Alaska Native, Asian or Pacific Islander (Asian alone and Native Hawaiian and other Pacific Islander alone combined), Hispanic, non-Hispanic Black (hereinafter, Black), non-Hispanic White (hereinafter, White), or 2 or more races (hereinafter, multiracial). The most prevalent racial and ethnic group was used as the reference (White, 50.1%).

#### Enabling Factors

Enabling factors included household income (≥400%, 200-<400%, 100-<200%, or <100% of the federal poverty level [FPL]), home ownership (owned or not owned), difficulty covering basics such as food or housing (never or rarely, or somewhat or very often), insurance type (private, public, or none), having a usual source of pediatric preventive care (yes or no), and having a personal doctor or nurse for the child (yes or no).

#### Need Factors

Need factors included perceived child health (excellent or very good, good, or fair or poor) and evaluated child health, indicating the number of health conditions present (0, 1, or ≥2 of the following 25: allergies, arthritis, asthma, autism, attention-deficit disorder or attention-deficit/hyperactivity disorder, anxiety, blindness, blood disorders, behavioral or conduct problems, concussion or brain injury, cystic fibrosis, cerebral palsy, Down syndrome, diabetes, depression, deafness, developmental delay, epilepsy, frequent or severe headaches including migraines, heart condition, intellectual disability, learning disability, speech or other language disorder, Tourette syndrome, and other genetic or inherited conditions). Each condition was noted to be present if the caregiver reported that a doctor or other health provider had ever told them that their child has the condition and—except for blindness, concussion, deafness, Down syndrome, and other genetic or inherited conditions—if the caregiver reported that the child currently has the condition.

#### Delayed or Missed Pediatric Preventive Care

The main study outcome was delayed or missed pediatric preventive care due to the COVID-19 pandemic (yes or no), as assessed using the following question: “During the past 12 months, did this child miss, delay, or skip any preventive check-ups because of the coronavirus pandemic?” Since the 2021 NSCH was fielded between June 25, 2021, and January 14, 2022,^25^ this question refers to delayed or missed pediatric preventive care between June 25, 2020, and January 14, 2022.

### Missing Data

A total of 50 892 respondents completed the 2021 NSCH (weighted response, 40.3%).^25^ Approximately 13.1% of observations had missing data, including for delayed or missed pediatric preventive care (4.5%), family structure (3.7%), difficulty covering basics (2.8%), insurance type (2.3%), usual source of pediatric preventive care (1.0%), having a personal doctor or nurse (0.8%), perceived child health (0.3%), and evaluated child health (8.8%). Frequencies of missing data for each variable by racial and ethnic group are presented in the eTable in Supplement 1. Children with missing data were notably less likely to be White, to live in a 2-parent household, to live in a house owned by their caregiver, to have a usual source of preventive care, to have a personal doctor or nurse, to have 2 or more health conditions, and to have delayed or missed preventive care. They were also substantially more likely to have caregivers with less than high school education, to have 4 or more children in the household, to have a main caregiver with good physical or mental health, to have a household income less than 100% of the FPL, and to have public or no insurance. Assuming that data were missing at random, we performed multiple imputation with chained equations to construct a complete data set, as has been widely reported in the literature.^28^ We performed 10 iterations using a random seed of 53421 by imputing missing data for all variables with missing data, using the remaining predisposing, enabling, and need factors as auxiliary variables.

### Statistical Analysis

First, we calculated weighted frequencies and 95% CIs for predisposing, enabling, and need factors in the overall cohort and by racial and ethnic group. Second, bivariate and multivariable Poisson regression analyses were used to estimate crude and adjusted prevalence ratios (PRs), respectively, and their 95% CIs for the associations between predisposing, enabling, and need factors and delayed or missed pediatric preventive care due to the COVID-19 pandemic in the overall cohort. Third, we performed multivariable Poisson regression stratified by racial and ethnic group, and we applied Bonferroni adjustment for multiple comparisons by dividing the significance level of .05 by the number of groups compared (ie, 6 racial and ethnic groups). All statistical analyses used multiple imputation with chained equations, were weighted to account for the complex NSCH survey design, and generated robust SEs. Accordingly, weighted data from this survey are representative of the population of noninstitutionalized children aged 0 to 17 years in the US. All statistical analyses were performed using Stata, version 17.0 (Stata/MP). All tests were 2 tailed; *P* < .05 was considered significant for the overall analysis, whereas *P* < .008 was considered significant for the stratified analysis. Data analysis was performed on February 21, 2023.

## Results

### Descriptive Analysis

Table 1 presents sample characteristics for the overall cohort and stratified by racial and ethnic group. Of the 50 892 respondents, 48.9% of children were female and 51.1% were male. Their mean (SD) age was 8.5 (5.3) years. With regard to race and ethnicity, 0.4% of children were American Indian or Alaska Native, 4.7% were Asian or Pacific Islander, 13.3% were Black, 25.8% were Hispanic, 50.1% were White, and 5.8% were multiracial. Overall, 27.6% of children had delayed or missed pediatric preventive care due to the COVID-19 pandemic (24.3% of American Indian or Alaska Native children, 26.5% of Asian or Pacific Islander children, 28.1% of Black children, 29.2% of Hispanic children, 26.3% of White children, and 32.1% of multiracial children).

**Table 1.** Sample Characteristics for the Overall Cohort and by Racial and Ethnic Group, 2021 National Survey of Children’s Health

Factor	Overall cohort (N = 50 892)	Racial and ethnic group, % (95% CI)					
		American Indian or Alaska Native (n = 316)	Asian or Pacific Islander (n = 3051)	Hispanic (n = 6916)	Non-Hispanic Black (n = 3291)	Non-Hispanic White (n = 33 565)	Multiracial (n = 3753)
**Predisposing**							
Child race and ethnicity							
American Indian or Alaska Native	0.4 (0.3-0.4)	NA	NA	NA	NA	NA	NA
Asian or Pacific Islander	4.7 (4.4-5.1)	NA	NA	NA	NA	NA	NA
Hispanic	25.8 (24.7-26.8)	NA	NA	NA	NA	NA	NA
Non-Hispanic Black	13.3 (12.6-14.0)	NA	NA	NA	NA	NA	NA
Non-Hispanic White	50.1 (49.1-51.0)	NA	NA	NA	NA	NA	NA
Multiracial	5.8 (5.4-6.2)	NA	NA	NA	NA	NA	NA
Child age, y							
0-2	15.4 (14.8-16.1)	6.9 (3.5-10.3)	16.1 (13.1-19.1)	14.3 (12.6-16.1)	12.6 (10.7-14.5)	16.1 (15.3-16.9)	21.3 (18.4-24.2)
3-5	16.5 (15.8-17.2)	13.0 (7.5-18.4)	17.2 (14.7-19.7)	16.8 (14.9-18.7)	18.0 (15.8-20.3)	15.9 (15.2-16.6)	17.2 (14.8-19.6)
6-8	16.1 (15.4-16.9)	16.5 (9.2-23.7)	17.6 (14.4-20.7)	14.6 (12.7-16.5)	16.2 (14.1-18.4)	16.9 (16.1-17.8)	14.7 (12.5-16.8)
9-11	17.2 (16.4-18.0)	19.7 (11.6-27.9)	14.3 (11.9-16.8)	17.5 (15.4-19.7)	17.1 (14.8-19.4)	17.4 (16.6-18.2)	16.9 (14.2-19.7)
12-14	17.7 (16.9-18.5)	20.3 (12.2-28.4)	15.7 (12.9-18.6)	18.6 (16.4-20.9)	18.5 (16.3-20.7)	17.3 (16.5-18.1)	17.1 (14.5-19.7)
15-17	16.9 (16.2-17.7)	23.6 (15.0-32.2)	19.0 (15.8-22.3)	18.1 (15.9-20.2)	17.5 (15.2-19.7)	16.4 (15.7-17.2)	12.9 (10.7-15.1)
Child sex							
Female	48.9 (47.9-49.9)	42.1 (32.8-51.4)	51.4 (47.6-55.3)	48.2 (45.4-50.9)	49.1 (46.2-52.1)	48.7 (47.7-49.8)	51.4 (48.0-54.7)
Male	51.1 (50.1-52.1)	57.9 (48.6-67.2)	48.6 (44.7-52.4)	51.8 (49.1-54.6)	50.9 (47.9-53.8)	51.3 (50.2-52.3)	48.6 (45.3-52.0)
Caregiver education							
High school or more	90.4 (89.5-91.3)	90.4 (81.7-99.2)	83.9 (80.0-87.8)	77.8 (75.2-80.5)	92.1 (90.1-94.2)	96.2 (95.5-96.9)	97.5 (96.1-98.9)
Less than high school	9.6 (8.7-10.5)	9.6 (8.4-18.3)	16.1 (12.2-20.0)	22.2 (19.5-24.8)	7.8 (5.8-9.9)	3.8 (3.1-4.5)	2.5 (1.1-3.9)
Family structure							
2 parents	71.0 (70.0-72.0)	51.9 (41.5-62.2)	73.6 (69.8-77.4)	66.0 (63.3-68.7)	45.0 (42.0-48.0)	80.6 (79.7-81.5)	69.4 (66.3-72.6)
Single mother	18.7 (17.9-19.6)	18.7 (11.1-26.2)	13.2 (10.5-15.9)	22.4 (20.1-24.7)	38.8 (35.8-41.8)	11.8 (11.1-12.6)	20.6 (17.9-23.4)
Single father	5.3 (4.8-5.8)	14.2 (6.1-22.3)	11.1 (7.9-14.3)	6.6 (4.9-8.2)	5.4 (4.2-6.6)	4.1 (3.6-4.5)	4.8 (3.2-6.4)
Grandparent or other	5.0 (4.5-5.5)	15.3 (7.5-23.1)	2.1 (1.3-2.9)	5.1 (3.7-6.4)	10.8 (8.8-12.8)	3.5 (3.1-4.0)	5.1 (3.6-6.6)
No. of children in household							
1	24.8 (24.0-25.5)	28.1 (19.8-36.5)	26.7 (23.7-29.7)	24.2 (22.0-26.3)	29.1 (26.8-31.4)	22.8 (22.0-23.5)	32.8 (29.5-36.0)
2	40.0 (39.1-41.0)	33.5 (23.9-43.1)	46.6 (42.8-50.5)	37.4 (34.7-40.1)	36.1 (33.2-39.0)	42.2 (41.1-43.2)	37.4 (34.2-40.5)
3	23.1 (22.1-24.0)	24.6 (16.2-32.9)	17.3 (13.9-20.7)	26.8 (24.2-29.4)	20.9 (18.4-23.5)	22.5 (21.5-23.5)	21.0 (18.1-23.8)
≥4	12.1 (11.4-12.9)	13.8 (7.7-19.9)	9.4 (6.4-12.4)	11.7 (9.8-13.5)	13.8 (11.5-16.2)	12.5 (11.7-13.4)	8.9 (6.9-10.9)
No. of children with special health care needs in household							
0	69.1 (68.2-70.0)	64.3 (55.2-73.4)	83.6 (80.5-86.7)	72.3 (69.9-74.7)	65.5 (62.7-68.3)	67.3 (66.3-68.3)	66.9 (63.7-70.0)
1	22.3 (21.5-23.1)	23.4 (15.2-31.7)	13.8 (10.9-16.6)	20.0 (18.0-22.1)	23.9 (21.5-26.3)	23.9 (23.0-24.8)	22.6 (19.8-25.4)
≥2	8.6 (8.0-9.2)	12.3 (6.8-17.8)	2.6 (1.3-4.0)	7.7 (6.2-9.2)	10.6 (8.6-12.6)	8.9 (8.3-9.4)	10.5 (8.5-12.5)
Physical or mental health of main caregiver							
Excellent or very good	55.1 (54.1-56.1)	41.4 (31.7-51.1)	62.8 (59.0-66.6)	51.9 (49.1-54.6)	46.7 (43.8-49.6)	58.8 (57.7-59.8)	52.6 (49.2-56.0)
Good	33.4 (32.5-34.4)	44.5 (34.9-54.2)	29.9 (26.4-33.4)	33.9 (31.3-36.4)	38.2 (35.3-41.1)	32.2 (31.2-33.2)	32.9 (29.8-36.0)
Fair or poor	11.5 (10.7-12.2)	14.1 (8.5-19.7)	7.3 (4.9-9.8)	14.3 (12.2-16.3)	15.2 (12.9-17.4)	9.0 (8.4-9.6)	14.5 (11.8-17.3)
**Enabling**							
Household income, % FPL							
≥400	31.8 (30.9-32.6)	23.6 (14.7-32.5)	38.5 (34.9-42.1)	18.5 (16.5-20.4)	15.3 (13.4-17.2)	41.4 (40.4-42.4)	40.4 (37.1-43.7)
200-<400	28.5 (27.7-29.4)	21.6 (14.9-28.3)	22.5 (19.6-25.5)	25.7 (23.3-28.1)	24.3 (21.9-26.8)	31.9 (30.9-32.9)	26.8 (24.0-29.6)
100-<200	20.7 (19.8-21.6)	23.2 (16.3-30.0)	17.7 (14.7-20.6)	26.9 (24.4-29.4)	27.4 (24.7-30.2)	16.3 (15.5-17.1)	17.8 (15.1-20.5)
<100	19.0 (18.1-19.9)	31.6 (21.6-41.6)	21.3 (17.3-25.3)	28.9 (26.3-31.5)	32.9 (30.1-35.7)	10.4 (9.7-11.1)	15.0 (12.4-17.6)
Home ownership							
Owned	72.8 (71.7-73.8)	64.9 (55.1-74.7)	75.4 (71.7-79.2)	60.8 (58.1-63.6)	52.6 (49.7-55.5)	84.0 (83.1-85.0)	72.9 (69.7-76.0)
Not owned	27.2 (26.3-28.3)	35.1 (25.3-44.9)	24.6 (20.8-28.3)	39.2 (36.4-41.9)	47.4 (44.5-50.3)	16.0 (15.0-16.9)	27.1 (24.0-30.3)
Difficulty covering basics							
Never or rarely	87.9 (87.1-88.6)	81.6 (74.9-88.3)	91.0 (88.7-93.2)	84.2 (82.0-86.3)	82.8 (80.4-85.2)	91.0 (90.3-91.7)	86.5 (84.2-88.8)
Somewhat or very often	12.1 (11.4-12.9)	18.4 (11.7-25.1)	9.0 (6.8-11.3)	15.8 (13.7-18.0)	17.2 (14.8-19.6)	9.0 (8.3-9.7)	13.5 (11.2-15.8)
Insurance type							
Private	61.7 (60.7-62.8)	46.2 (36.6-55.8)	66.0 (62.0-70.0)	45.4 (42.7-48.1)	43.4 (40.5-46.4)	74.0 (73.0-75.1)	67.2 (63.9-70.4)
Public	30.8 (29.8-31.8)	35.3 (26.5-44.2)	25.3 (21.5-29.0)	42.4 (39.6-45.2)	49.8 (46.8-52.8)	20.6 (19.7-21.6)	28.4 (25.3-31.6)
None	7.5 (6.8-8.2)	18.5 (9.0-28.0)	8.7 (6.1-11.4)	12.2 (10.1-14.2)	6.8 (5.1-8.4)	5.4 (4.7-6.0)	4.4 (2.9-5.9)
Usual source of pediatric preventive care							
Yes	89.0 (88.2-89.8)	85.3 (76.4-94.3)	81.3 (77.9-84.7)	80.5 (78.1-83.0)	86.9 (84.8-89.1)	94.2 (93.6-94.8)	93.3 (91.6-95.1)
No	11.0 (10.2-11.8)	14.7 (5.7-23.6)	18.7 (15.3-22.1)	19.5 (17.0-21.9)	13.1 (10.9-15.2)	5.8 (5.2-6.4)	6.7 (4.9-8.4)
Personal doctor or nurse for child							
Yes	70.9 (69.9-71.8)	63.1 (53.4-72.8)	65.2 (61.4-69.0)	61.5 (58.8-64.3)	66.8 (64.0-69.6)	76.9 (76.0-77.9)	73.7 (70.6-76.7)
No	29.1 (28.2-30.1)	36.9 (27.2-46.5)	34.8 (31.0-38.6)	38.5 (35.7-41.2)	33.2 (30.4-36.0)	23.1 (22.1-24.0)	26.3 (23.3-29.4)
**Need**							
Perceived child health							
Excellent or very good	89.8 (89.1-90.6)	87.2 (81.7-92.6)	89.6 (86.9-92.2)	84.8 (82.6-87.1)	85.2 (82.9-87.5)	93.4 (92.9-93.9)	92.5 (90.9-94.2)
Good	8.3 (7.6-8.9)	11.9 (6.5-17.2)	8.6 (6.2-11.1)	12.1 (10.1-14.0)	11.6 (9.6-13.5)	5.6 (5.2-6.1)	6.1 (4.6-7.6)
Fair or poor	1.9 (1.5-2.3)	0.9 (0.1-1.9)	1.8 (0.6-3.1)	3.1 (1.8-4.3)	3.2 (1.9-4.5)	1.0 (0.8-1.3)	1.3 (0.7-2.0)
No. of health conditions^a^							
0	60.7 (59.8-61.7)	47.3 (36.8-57.8)	77.1 (73.6-80.5)	63.1 (60.4-65.8)	59.1 (56.1-62.1)	59.0 (58.0-60.1)	56.8 (53.4-60.2)
1	19.4 (18.6-20.2)	23.0 (13.4-32.6)	15.3 (12.2-18.5)	19.0 (16.7-21.3)	18.1 (15.8-20.6)	20.2 (19.4-21.0)	19.7 (17.0-22.4)
≥2	19.9 (19.1-20.7)	29.7 (19.9-39.6)	7.6 (5.7-9.4)	18.1 (16.1-20.1)	22.8 (20.3-25.2)	20.8 (19.9-21.6)	23.6 (20.6-26.5)
**Delayed or missed pediatric preventive care**							
No	72.4 (71.5-73.3)	75.7 (67.8-83.6)	73.5 (70.2-76.9)	70.8 (68.3-73.4)	71.9 (69.1-74.8)	73.7 (72.8-74.6)	70.0 (64.6-71.4)
Yes	27.6 (26.7-28.5)	24.3 (16.4-32.2)	26.5 (23.1-29.8)	29.2 (26.6-31.7)	28.1 (25.2-30.9)	26.3 (25.4-27.2)	32.1 (28.6-35.4)

Abbreviations: FPL, federal poverty level; NA, not applicable.

^a^ Includes the following conditions: allergies, arthritis, asthma, autism (eg, autism spectrum disorder, Asperger syndrome, or pervasive developmental disorder), attention-deficit disorder or attention-deficit/hyperactivity disorder, anxiety, blindness, blood disorders (eg, sickle cell disease, thalassemia, or hemophilia), behavioral or conduct problems, concussion or brain injury, cystic fibrosis, cerebral palsy, Down syndrome, diabetes, depression, deafness, developmental delay, epilepsy, frequent or severe headaches including migraines, heart condition, intellectual disability, learning disability, speech or other language disorder, Tourette syndrome, and other genetic or inherited conditions.

### Overall Analysis

Table 2 presents the crude and adjusted analyses for the overall cohort. Notable risk factors for delayed or missed pediatric preventive care included predisposing factors such as race (Asian or Pacific Islander vs non-Hispanic White [PR, 1.16 (95% CI, 1.02-1.32)] and multiracial vs non-Hispanic White [PR, 1.23 (95% CI, 1.11-1.37)]), Hispanic ethnicity (PR, 1.19 [95% CI, 1.09-1.31]), older age (6-8 years vs 0-2 years [PR, 1.73 (95% CI, 1.52-1.97)]), and fair or poor vs excellent or very good caregiver health (PR, 1.27 [95% CI, 1.14-1.41]); enabling factor difficulty covering basics somewhat or very often vs never or rarely (PR, 1.41 [95% CI, 1.28-1.55]); and need factors such as fair or poor vs excellent or very good perceived child health (PR, 1.35 [95% CI, 1.06-1.72]) and 2 or more vs 0 health conditions (PR, 1.20 [95% CI, 1.09-1.32]). Notable protective factors included predisposing factor single-father vs 2-parent household (PR, 0.74 [95% CI, 0.61-0.89]) and enabling factors such as lower household income (<100% vs ≥400% FPL [PR, 0.79 (95% CI, 0.69-0.91)]) and no usual source of preventive care (PR, 0.84 [95% CI, 0.72-0.99]). Additional risk and protective factors are presented in Table 2.

**Table 2.** Bivariate and Multivariable Associations Between Predisposing, Enabling, and Need Factors With COVID-19–Related Delayed or Missed Pediatric Preventive Care in the Overall Cohort

Factor	Overall cohort (N = 50 892)	
	Crude PR (95% CI)	Adjusted PR (95% CI)^a^
**Predisposing**		
Child race and ethnicity		
American Indian or Alaska Native	0.92 (0.67-1.27)	0.91 (0.66-1.24)
Asian or Pacific Islander	1.01 (0.88-1.15)	1.16 (1.02-1.32)^b^
Hispanic	1.11 (1.01-1.22)^b^	1.19 (1.09-1.31)^b^
Non-Hispanic Black	1.07 (0.96-1.19)	1.09 (0.98-1.22)
Non-Hispanic White	1 [Reference]	1 [Reference]
Multiracial	1.22 (1.09-1.37)^b^	1.23 (1.11-1.37)^b^
Child age, y		
0-2	1 [Reference]	1 [Reference]
3-5	1.60 (1.40-1.82)^b^	1.53 (1.35-1.75)^b^
6-8	1.84 (1.61-2.10)^b^	1.73 (1.52-1.97)^b^
9-11	1.82 (1.59-2.08)^b^	1.71 (1.50-1.96)^b^
12-14	1.74 (1.52-1.99)^b^	1.61 (1.40-1.83)^b^
15-17	1.72 (1.50-1.96)^b^	1.59 (1.39-1.83)^b^
Family structure		
2 parents	1 [Reference]	1 [Reference]
Single mother	1.05 (0.95-1.15)	0.95 (0.86-1.05)
Single father	0.71 (0.59-0.86)^b^	0.74 (0.61-0.89)^b^
Grandparent or other	0.95 (0.79-1.14)	0.92 (0.77-1.10)
No. of children in household		
1	1 [Reference]	1 [Reference]
2	1.08 (1.00-1.17)	1.06 (0.98-1.15)
3	1.10 (1.00-1.21)	1.08 (0.98-1.19)
≥4	1.14 (1.01-1.28)^b^	1.15 (1.02-1.31)^b^
Main caregiver physical or mental health		
Excellent or very good	1 [Reference]	1 [Reference]
Good	1.24 (1.16-1.34)^b^	1.18 (1.10-1.27)^b^
Fair or poor	1.49 (1.35-1.65)^b^	1.27 (1.14-1.41)^b^
**Enabling**		
Household income, % FPL		
≥400	1 [Reference]	1 [Reference]
200-<400	0.93 (0.86-1.00)	0.87 (0.80-0.94)^b^
100-<200	0.96 (0.88-1.06)	0.84 (0.75-0.95)^b^
<100	0.91 (0.82-1.01)	0.79 (0.69-0.91)^b^
Difficulty covering basics		
Never or rarely	1 [Reference]	1 [Reference]
Somewhat or very often	1.48 (1.35-1.62)^b^	1.41 (1.28-1.55)^b^
Usual source of pediatric preventive care		
Yes	1 [Reference]	1 [Reference]
No	0.77 (0.66-0.90)^b^	0.84 (0.72-0.99)^b^
Personal doctor or nurse for child		
Yes	1 [Reference]	1 [Reference]
No	0.84 (0.77-0.91)^b^	0.90 (0.83-0.98)^b^
**Need**		
Perceived child health		
Excellent or very good	1 [Reference]	1 [Reference]
Good	1.38 (1.23-1.55)^b^	1.17 (1.04-1.31)^b^
Fair or poor	1.65 (1.28-2.11)^b^	1.35 (1.06-1.72)^b^
No. of health conditions^c^		
0	1 [Reference]	1 [Reference]
1	1.26 (1.16-1.37)^b^	1.12 (1.03-1.23)^b^
≥2	1.54 (1.43-1.66)^b^	1.20 (1.09-1.32)^b^

Abbreviations: FPL, federal poverty level; PR, prevalence ratio.

^a^ Multivariable analysis was adjusted for all predisposing, enabling, and need factors presented as well as for the following variables that were not significant at *P* < .05 and thus not presented: child sex, caregiver education, number of children with special health care needs in the household, home ownership, and insurance.

^b^ Indicates significance at *P* < .05.

^c^ Includes the following conditions: allergies, arthritis, asthma, autism (eg, autism spectrum disorder, Asperger syndrome, or pervasive developmental disorder), attention-deficit disorder or attention-deficit/hyperactivity disorder, anxiety, blindness, blood disorders (eg, sickle cell disease, thalassemia, or hemophilia), behavioral or conduct problems, concussion or brain injury, cystic fibrosis, cerebral palsy, Down syndrome, diabetes, depression, deafness, developmental delay, epilepsy, frequent or severe headaches including migraines, heart condition, intellectual disability, learning disability, speech or other language disorder, Tourette syndrome, and other genetic or inherited conditions.

### Stratified Analysis

In adjusted analysis stratified by racial and ethnic group (Table 3), notable risk factors among multiracial children included predisposing and need factors such as age 9 to 11 years (vs 0-2 years [PR, 1.73 (95% CI, 1.16-2.57)]) and 2 or more vs 0 health conditions (PR, 1.54 [95% CI, 1.14-2.08]). Among Black participants, notable predisposing and enabling factors included age 6 to 8 years (vs 0-2 years [PR, 1.90 (95% CI, 1.23-2.92)]) and difficulty covering basics somewhat or very often vs never or rarely (PR, 1.68 [95% CI, 1.35-2.09]). Notable risk factors among non-Hispanic White participants included predisposing factors such as older age (9-11 years vs 0-2 years [PR, 2.05 (95% CI, 1.78-2.37)]), 4 or more children vs 1 child in the household (PR, 1.22 [95% CI, 1.07-1.39]), and fair or poor vs excellent or very good caregiver health (PR, 1.32 [95% CI, 1.18-1.47]); enabling factor difficulty covering basics somewhat or very often vs never or rarely (PR, 1.36 [95% CI, 1.22-1.52]); and need factors such as good vs excellent or very good perceived child health (PR, 1.19 [95% CI, 1.06-1.34]) and 2 or more vs 0 health conditions (PR, 1.25 [95% CI, 1.12-1.38]). Among multiracial children, notable protective factors included enabling factors—namely, lower household income (<100% vs ≥400% FPL [PR, 0.52 (95% CI, 0.35-0.79)]). Additional risk factors are presented in Table 3.

**Table 3.** Multivariable Associations Between Predisposing, Enabling, and Need Factors With COVID-19–Related Delayed or Missed Pediatric Preventive Care by Racial and Ethnic Group

Factor	Adjusted PR (95% CI) (N = 50 892)^a^					
	American Indian or Alaska Native (n = 316)	Asian or Pacific Islander (n = 3051)	Hispanic (n = 6916)	Non-Hispanic Black (n = 3291)	Non-Hispanic White (n = 33 565)	Multiracial (n = 3753)
**Predisposing**						
Child age, y						
0-2	1 [Reference]	1 [Reference]	1 [Reference]	1 [Reference]	1 [Reference]	1 [Reference]
3-5	1.16 (0.29-4.56)	1.45 (0.87-2.42)	1.20 (0.88-1.63)	1.57 (1.02-2.41)	1.81 (1.57-2.09)^b^	1.46 (0.99-2.14)
6-8	1.58 (0.45-5.76)	1.88 (1.10-3.22)	1.31 (0.95-1.82)	1.90 (1.23-2.92)^b^	2.01 (1.74-2.33)^b^	1.54 (1.05-2.25)
9-11	2.18 (0.57-8.28)	1.67 (0.97-2.90)	1.25 (0.90-1.75)	1.66 (1.06-2.59)	2.05 (1.78-2.37)^b^	1.73 (1.16-2.57)^b^
12-14	1.17 (0.29-4.66)	1.40 (0.81-2.41)	1.31 (0.95-1.81)	1.73 (1.12-2.67)	1.84 (1.59-2.13)^b^	1.56 (1.06-2.29)
15-17	0.92 (0.25-3.43)	1.57 (0.95-2.60)	1.32 (0.94-1.85)	1.57 (1.03-2.39)	1.83 (1.59-2.12)^b^	1.50 (1.00-2.25)
No. of children in household						
1	1 [Reference]	1 [Reference]	1 [Reference]	1 [Reference]	1 [Reference]	1 [Reference]
2	1.10 (0.56-2.16)	1.12 (0.85-1.49)	1.04 (0.83-1.29)	1.04 (0.86-1.42)	1.10 (1.01-1.19)	0.92 (0.73-1.16)
3	1.23 (0.59-2.57)	0.98 (0.64-1.50)	1.06 (0.83-1.36)	1.04 (0.77-1.40)	1.13 (1.01-1.26)	1.00 (0.75-1.34)
≥4	0.31 (0.06-1.47)	0.60 (0.33-1.09)	1.17 (0.83-1.64)	1.22 (0.88-1.69)	1.22 (1.07-1.39)^b^	1.05 (0.68-1.62)
Main caregiver’s physical or mental health						
Excellent or very good	1 [Reference]	1 [Reference]	1 [Reference]	1 [Reference]	1 [Reference]	1 [Reference]
Good	1.46 (0.77-2.75)	1.04 (0.79-1.36)	1.25 (1.03-1.51)	1.25 (1.00-1.57)	1.14 (1.05-1.23)^b^	1.16 (0.94-1.44)
Fair or poor	1.52 (0.67-3.46)	1.04 (0.67-1.61)	1.29 (1.00-1.66)	1.22 (0.90-1.65)	1.32 (1.18-1.47)^b^	1.34 (1.03-1.75)
**Enabling**						
Household income, % FPL						
≥400	1 [Reference]	1 [Reference]	1 [Reference]	1 [Reference]	1 [Reference]	1 [Reference]
200-<400	1.36 (0.65-2.87)	0.77 (0.56-1.07)	0.86 (0.68-1.09)	0.84 (0.61-1.16)	0.92 (0.85-0.99)	0.69 (0.55-0.88)^b^
100-<200	1.06 (0.40-2.80)	0.66 (0.40-1.09)	0.84 (0.64-1.10)	0.90 (0.62-1.32)	0.87 (0.76-0.99)	0.60 (0.42-0.85)^b^
<100	0.94 (0.38-2.29)	0.50 (0.28-0.87)	0.82 (0.61-1.11)	0.78 (0.52-1.16)	0.87 (0.73-1.03)	0.52 (0.35-0.79)^b^
Difficulty covering basics						
Never or rarely	1 [Reference]	1 [Reference]	1 [Reference]	1 [Reference]	1 [Reference]	1 [Reference]
Somewhat or very often	2.22 (1.21-4.09)	1.53 (1.07-2.20)	1.32 (1.05-1.64)	1.68 (1.35-2.09)^b^	1.36 (1.22-1.52)^b^	1.32 (1.03-1.70)
**Need**						
Perceived child health						
Excellent or very good	1 [Reference]	1 [Reference]	1 [Reference]	1 [Reference]	1 [Reference]	1 [Reference]
Good	0.58 (0.17-1.98)	1.22 (0.83-1.77)	1.10 (0.83-1.44)	1.31 (1.01-1.72)	1.19 (1.06-1.34)^b^	1.12 (0.76-1.65)
Fair or poor	0.84 (0.12-5.99)	1.38 (0.64-2.95)	1.32 (0.81-2.15)	1.57 (0.99-2.49)	1.22 (0.86-1.74)	1.56 (0.93-2.61)
No. of health conditions^c^						
0	1 [Reference]	1 [Reference]	1 [Reference]	1 [Reference]	1 [Reference]	1 [Reference]
1	0.85 (0.44-1.66)	0.97 (0.72-1.31)	0.99 (0.78-1.25)	1.17 (0.86-1.59)	1.15 (1.05-1.26)^b^	1.52 (1.18-1.95)^b^
≥2	0.95 (0.40-2.22)	0.89 (0.59-1.35)	1.04 (0.79-1.37)	1.26 (0.93-1.70)	1.25 (1.12-1.38)^b^	1.54 (1.14-2.08)^b^

Abbreviations: FPL, federal poverty level; PR, prevalence ratio.

^a^ Multivariable analysis was adjusted for all predisposing, enabling, and need factors presented and for the following variables that were not significant at *P* < .008 and thus not presented: child sex, caregiver education, family structure, number of children with special health care needs in the household, home ownership, insurance, usual source of preventive care, and personal doctor or nurse for the child.

^b^ Indicates significance at *P* < .008, using Bonferroni adjustment for multiple comparison.

^c^ Includes the following conditions: allergies, arthritis, asthma, autism (eg, autism spectrum disorder, Asperger syndrome, or pervasive developmental disorder), attention-deficit disorder or attention-deficit/hyperactivity disorder, anxiety, blindness, blood disorders (eg, sickle cell disease, thalassemia, or hemophilia), behavioral or conduct problems, concussion or brain injury, cystic fibrosis, cerebral palsy, Down syndrome, diabetes, depression, deafness, developmental delay, epilepsy, frequent or severe headaches including migraines, heart condition, intellectual disability, learning disability, speech or other language disorder, Tourette syndrome, and other genetic or inherited conditions.

## Discussion

The results of this study suggest that 27.6% of US children aged 0 to 17 years had delayed or missed preventive care due to the COVID-19 pandemic, which is consistent with the limited available research in this area.^3,4,5^ We observed that several predisposing, enabling, and need factors were associated with delayed or missed pediatric preventive care, with differences by racial and ethnic group.

### Risk and Protective Factors in the Overall Cohort

In the adjusted analysis for the overall cohort, predisposing factors such as race and ethnicity and child age were associated with delayed or missed pediatric preventive care. Asian or Pacific Islander, Hispanic, and multiracial groups were notably more likely to have delayed or missed preventive care. Results from previous studies have been mixed, reporting increased likelihood among multiracial children^5^ and Hispanic children.^6^ Prepandemic research reported that Hispanic children were less likely to meet preventive visit recommendations,^29^ suggesting that the pandemic may have sustained or exacerbated barriers for some groups while potentially creating new barriers for others.

Our finding that older children were more likely to have delayed or missed preventive care is consistent with previous research^6^ and may reflect caregiver prioritization of well-child visits, during which vaccinations are typically administered.^30^ In addition, our observation that children in households with a higher number of children were more likely to have delayed or missed preventive care is consistent with prior studies^4,5,6^ and may reflect competing needs. For instance, caregivers with a higher number of children were more likely to report barriers such as COVID-19 exposure or COVID-19 illness in the household.^5^ Children in a single-father household were less likely to have delayed or missed preventive care. This is in contrast with prepandemic NSCH data^31^ but consistent with previous research during the pandemic^4^ and may reflect increased fear of COVID-19 exposure during pediatric visits among mothers vs fathers.^5^ Notably, we observed that poor caregiver health, which was not previously examined in a similar context, was a risk factor for delayed or missed preventive care, possibly reflecting concern over COVID-19 exposure or other accessibility barriers.^32,33^

Enabling factors were also associated with preventive care use in the overall cohort. Consistent with previous research,^4,5^ we observed that difficulty covering basics was a risk factor for delayed or missed preventive care, whereas lower household income was a protective factor. Although financial hardship may reduce affordability of care, parents in higher-income households were more likely to be concerned over COVID-19 exposure during pediatric visits.^5^ Notably, we observed that not having a medical home—which has not been previously examined in a similar context, to our knowledge—was a protective factor for delayed or missed preventive care. Although children with a medical home receive more preventive care,^33,34^ our findings suggest that they also experienced more disruptions to care during the pandemic.

Finally, we observed that need factors such as poor perceived or evaluated child health—which were not previously examined in a similar context, to our knowledge—were risk factors for delayed or missed preventive care in the overall cohort. Although children with poorer health receive more preventive care,^35^ our findings suggest that they also experienced more disruptions to care during the pandemic.

### Risk and Protective Factors by Racial and Ethnic Group

In the adjusted analysis stratified by racial and ethnic group, we observed that older age was the only predisposing risk factor among Black and multiracial children. Among White children, predisposing factors associated with delayed or missed preventive care were similar to the overall cohort except for single-father households, which was no longer statistically significant after Bonferroni adjustment. With regard to enabling factors, we observed that lower income was a protective factor among multiracial children. Difficulty covering basics was a risk factor among Black and White children, whereas it was no longer statistically significant after Bonferroni adjustment among other racial and ethnic groups. With regard to need factors, we observed that the presence of 1 or more health conditions was a risk factor among multiracial children. Among White children, need factors associated with delayed or missed preventive care were similar to the overall cohort.

Although we identified several risk factors among White children, we identified a few risk and protective factors among multiracial and Black children and none among American Indian or Alaska Native, Asian or Pacific Islander, and Hispanic children, particularly after Bonferroni adjustment. It is possible that the latter may experience barriers to care that were not examined in this study, such as transportation challenges, limited appointment availability in the area, or long waiting times.^20^ In previous research, the most frequently reported reasons for delayed or missed pediatric preventive care included fear of COVID-19 exposure, reported among Asian, Black, and White individuals; limited appointments, reported among Hispanic, White, and multiracial individuals; and clinics being closed, reported among Black and Hispanic individuals; no such studies examined American Indian or Alaska Native individuals separately.^5,6^

### Implications

Our finding that more than one-fourth of US children experienced delayed or missed preventive care due to the COVID-19 pandemic highlights the need for targeted outreach efforts to promote catch-up visits, particularly among high-risk groups, including Asian or Pacific Islander, Hispanic, and multiracial children. Several predisposing, enabling, and need factors were associated with delayed or missed preventive care. Specifically, our findings point to the potential importance of social programs and policies aiming to address financial hardship. We also identified factors that may guide targeted interventions among different racial and ethnic groups, including older age, higher income, and poor health among multiracial children; older age and financial hardship among Black children; and older age, a high number of children in the household, poor caregiver health, financial hardship, and poor health among White children.

### Strengths and Limitations

This study expands on the scarce body of research in this area by (1) using the Andersen behavioral model of health services use to examine a wide variety of predisposing, enabling, and need factors in relation to delayed or missed preventive care due to the COVID-19 pandemic in a nationally representative sample of US children and (2) examining such associations by racial and ethnic group. Strengths of this study include its population-based study design, enabling generalization to the US population; its large sample size, allowing ample statistical power to identify associations; the availability of information on a variety of predisposing, enabling, and need factors; and stratified analysis by racial and ethnic group, enabling the identification of race and ethnicity–specific associations.

Our study also has some limitations. First, survey responses were self-reported by the child’s parent or caregiver and may be subject to recall or social desirability bias, which may have underestimated our findings. Indeed, parental reports have been shown to overestimate child health care use compared with administrative and billing data.^36^ Second, the NSCH does not differentiate between delayed vs missed preventive care, which may differentially affect morbidity and mortality. Third, our results may be underestimated if caregivers did not consider that preventive care was delayed or missed because they did not follow pediatric preventive care schedules. Fourth, we cannot demonstrate temporality or causality for the associations under study because of the cross-sectional nature of the data.

## Conclusions

In this cross-sectional study, more than one-fourth of children had delayed or missed preventive care due to the COVID-19 pandemic. Our findings highlight risk and protective factors among different racial and ethnic groups and may guide targeted interventions aiming to promote timely pediatric preventive care. Future studies should further explore the risk and protective factors for delayed or missed pediatric preventive care, particularly among racial and ethnic minority individuals.

## Notes

Supplement 1. **eTable.** Frequency of Missing Data for Predisposing, Enabling, and Need Factors and Delayed or Missed Preventive Care, by Racial and Ethnic Group (N = 50 892)

Supplement 2.
Data Sharing Statement

## References

[1] Lazzerini M, Barbi E, Apicella A, Marchetti F, Cardinale F, Trobia G. Delayed access or provision of care in Italy resulting from fear of COVID-19. Lancet Child Adolesc Health. 2020;4(5):e10-e11. doi:10.1016/S2352-4642(20)30108-5 32278365 PMC7146704

[2] Dinleyici EC, Borrow R, Safadi MAP, van Damme P, Munoz FM. Vaccines and routine immunization strategies during the COVID-19 pandemic. Hum Vaccin Immunother. 2021;17(2):400-407. doi:10.1080/21645515.2020.1804776 32845739 PMC7899627

[3] California Department of Health Care Services. 2021 Preventive Services Report. 2022. Accessed January 6, 2023. https://www.dhcs.ca.gov/Documents/MCQMD/2020-21-Preventive-Services-Report-0603.pdf

[4] Lebrun-Harris LA, Sappenfield OR, Warren MD. Missed and delayed preventive health care visits among US children due to the COVID-19 pandemic. Public Health Rep. 2022;137(2):336-343. doi:10.1177/00333549211061322 34969335 PMC8900224

[5] Nguyen KH, Nguyen K, Lekshmi D, Corlin L, Niska RW. Delays in children’s preventive health services during the COVID-19 pandemic. Fam Med. 2022;54(5):350-361. doi:10.22454/FamMed.2022.922801 35536620

[6] Teasdale CA, Borrell LN, Shen Y, . Missed routine pediatric care and vaccinations in US children during the first year of the COVID-19 pandemic. Prev Med. 2022;158:107025. doi:10.1016/j.ypmed.2022.107025 35318030 PMC8933962

[7] Patel Murthy B, Zell E, Kirtland K, . Impact of the COVID-19 pandemic on administration of selected routine childhood and adolescent vaccinations—10 U.S. jurisdictions, March-September 2020. MMWR Morb Mortal Wkly Rep. 2021;70(23):840-845. doi:10.15585/mmwr.mm7023a2 34111058 PMC8191867

[8] Shapiro GK, Gottfredson N, Leask J, . COVID-19 and missed or delayed vaccination in 26 middle- and high-income countries: an observational survey. Vaccine. 2022;40(6):945-952. doi:10.1016/j.vaccine.2021.12.041 35039193 PMC8687753

[9] Ciacchini B, Tonioli F, Marciano C, . Reluctance to seek pediatric care during the COVID-19 pandemic and the risks of delayed diagnosis. Ital J Pediatr. 2020;46(1):87. doi:10.1186/s13052-020-00849-w 32600464 PMC7322712

[10] Meher BK, Panda I, Mishra NR, Das L, Sahu B. The impact of COVID-19 on pediatric healthcare utilization and disease dynamics: an observational study from Western Odisha. Cureus. 2022;14(7):e27006. doi:10.7759/cureus.27006 36000109 PMC9390950

[11] Alcocer Alkureishi L. Acute delays in pediatric primary care delivery due to the pandemic. Pediatr Ann. 2022;51(10):e374-e375. doi:10.3928/19382359-20220902-01 36215087

[12] Cherubini V, Gohil A, Addala A, . Unintended consequences of coronavirus disease-2019: remember general pediatrics. J Pediatr. 2020;223:197-198. doi:10.1016/j.jpeds.2020.05.004 32437758 PMC7207102

[13] Cousino MK, Pasquali SK, Romano JC, . Impact of the COVID-19 pandemic on CHD care and emotional wellbeing. Cardiol Young. 2021;31(5):822-828. doi:10.1017/S1047951120004758 33308334 PMC7783141

[14] Dermott JA, Kim DJ, Lebel DE. The impact of COVID-19 on idiopathic scoliosis referrals: cause for concern. Spine Deform. 2021;9(6):1501-1507. doi:10.1007/s43390-021-00418-z 34596888 PMC8485111

[15] Offenbacher R, Knoll MA, Loeb DM. Delayed presentations of pediatric solid tumors at a tertiary care hospital in the Bronx due to COVID-19. Pediatr Blood Cancer. 2021;68(2):e28615. doi:10.1002/pbc.28615 32725878

[16] Snapiri O, Rosenberg Danziger C, Krause I, . Delayed diagnosis of paediatric appendicitis during the COVID-19 pandemic. Acta Paediatr. 2020;109(8):1672-1676. doi:10.1111/apa.15376 32460364 PMC7283758

[17] Diskin C, Orkin J, Agarwal T, Parmar A, Friedman JN. The secondary consequences of the COVID-19 pandemic in hospital pediatrics. Hosp Pediatr. 2021;11(2):208-212. doi:10.1542/hpeds.2020-002477 33436416

[18] Lebrun-Harris LA, Canto MT, Vodicka P, Mann MY, Kinsman SB. Oral health among children and youth with special health care needs. Pediatrics. 2021;148(2):e2020025700. doi:10.1542/peds.2020-025700 34290133

[19] Swope CB, Hernández D. Housing as a determinant of health equity: a conceptual model. Soc Sci Med. 2019;243:112571. doi:10.1016/j.socscimed.2019.112571 31675514 PMC7146083

[20] Caraballo C, Ndumele CD, Roy B, . Trends in racial and ethnic disparities in barriers to timely medical care among adults in the US, 1999 to 2018. JAMA Health Forum. 2022;3(10):e223856. doi:10.1001/jamahealthforum.2022.3856 36306118 PMC9617175

[21] Boserup B, McKenney M, Elkbuli A. Disproportionate impact of COVID-19 pandemic on racial and ethnic minorities. Am Surg. 2020;86(12):1615-1622. doi:10.1177/0003134820973356 33231496 PMC7691116

[22] Data Resource Center for Child and Adolescent Health. 2021 National Survey of Children’s Health, Stata indicator dataset. Child and Adolescent Health Measurement Initiative. 2022. Accessed December 23, 2022. https://www.childhealthdata.org/

[23] Ghandour RM, Jones JR, Lebrun-Harris LA, . The design and implementation of the 2016 National Survey of Children’s Health. Matern Child Health J. 2018;22(8):1093-1102. doi:10.1007/s10995-018-2526-x 29744710 PMC6372340

[24] Data Resource Center for Child and Adolescent Health. Fast facts: 2020-2021 National Survey of Children’s Health combined dataset. Child and Adolescent Health Measurement Initiative. 2022. Accessed February 3, 2023. https://www.childhealthdata.org/docs/default-source/nsch-docs/2020-2021-nsch-fast-facts_cahmi_10-1-22.pdf?sfvrsn=78645c17_10

[25] US Census Bureau. 2021 National Survey of Children’s Health methodology report. Accessed February 7, 2023. https://www2.census.gov/programs-surveys/nsch/technical-documentation/methodology/2021-NSCH-Methodology-Report.pdf

[26] Andersen RM. Revisiting the behavioral model and access to medical care: does it matter? J Health Soc Behav. 1995;36(1):1-10. doi:10.2307/2137284 7738325

[27] Babitsch B, Gohl D, von Lengerke T. Re-revisiting Andersen’s behavioral model of health services use: a systematic review of studies from 1998-2011. Psychosoc Med. 2012;9:Doc11. 23133505 10.3205/psm000089 PMC3488807

[28] Pedersen AB, Mikkelsen EM, Cronin-Fenton D, . Missing data and multiple imputation in clinical epidemiological research. Clin Epidemiol. 2017;9:157-166. doi:10.2147/CLEP.S129785 28352203 PMC5358992

[29] Ronsaville DS, Hakim RB. Well child care in the United States: racial differences in compliance with guidelines. Am J Public Health. 2000;90(9):1436-1443. doi:10.2105/AJPH.90.9.1436 10983203 PMC1447611

[30] Wolf ER, O’Neil J, Pecsok J, . Caregiver and clinician perspectives on missed well-child visits. Ann Fam Med. 2020;18(1):30-34. doi:10.1370/afm.2466 31937530 PMC7227475

[31] Data Resource Center for Child and Adolescent Health. 2019 National Survey of Children’s Health data query. Child and Adolescent Health Measurement Initiative. 2022. Accessed May 19, 2023. https://www.childhealthdata.org/browse/survey/results?q=8210&r=1&g=837

[32] Kullgren JT, McLaughlin CG, Mitra N, Armstrong K. Nonfinancial barriers and access to care for U.S. adults. Health Serv Res. 2012;47(1 Pt 2):462-485. doi:10.1111/j.1475-6773.2011.01308.x 22092449 PMC3393009

[33] Callison K, Ward J. Associations between individual demographic characteristics and involuntary health care delays as a result of COVID-19. Health Aff (Millwood). 2021;40(5):837-843. doi:10.1377/hlthaff.2021.00101 33881908

[34] Data Resource Center for Child and Adolescent Health. 2021 National Survey of Children’s Health: sampling and survey administration. Child and Adolescent Health Measurement Initiative. 2022. Accessed May 19, 2023. https://www.childhealthdata.org/browse/survey/results?q=9762&r=1&g=1053

[35] Berdahl T, Biener A, McCormick MC, Guevara JP, Simpson L. Annual report on children’s healthcare: healthcare access and utilization by obesity status in the United States. Acad Pediatr. 2020;20(2):175-187. doi:10.1016/j.acap.2019.11.020 31843708

[36] Chung PJ, Lee TC, Morrison JL, Schuster MA. Preventive care for children in the United States: quality and barriers. Annu Rev Public Health. 2006;27:491-515. doi:10.1146/annurev.publhealth.27.021405.102155 16533127

