# Supplementary material for: Racial and Ethnic Differences in Factors Associated With Delayed or Missed Pediatric Preventive Care in the US Due to the COVID-19 Pandemic
Source: JAMA Netw Open. 2023 Jul 10;6(7):e2322588. doi: 10.1001/jamanetworkopen.2023.22588 (PMC10334219; doi:10.1001/jamanetworkopen.2023.22588)
Supplement: Supplement 1. — eTable. Frequency of Missing Data for Predisposing, Enabling, and Need Factors and Delayed or Missed Preventive Care, by Racial and Ethnic Group (N = 50 892) [file jamanetwopen-e2322588-s001.pdf]

Supplementary Online Content

Tabet M, Kirby RS, Xaverius P. Racial and ethnic differences in factors associated with delayed or missed pediatric preventive care in the US due to the COVID-19 pandemic. *JAMA Netw Open*. 2023;6(7):e2322588. doi:10.1001/jamanetworkopen.2023.22588

eTable. Frequency of Missing Data for Predisposing, Enabling, and Need Factors and Delayed or Missed Preventive Care, by Racial and Ethnic Group (N = 50 892)

This supplementary material has been provided by the authors to give readers additional information about their work.

© 2023 Tabet M et al. *JAMA Network Open*.

eTable. Frequency of Missing Data for Predisposing, Enabling, and Need Factors and Delayed or Missed Preventive Care, by Racial and Ethnic Group (N = 50 892)

	American Indian/Alaska Native	Asian/Pacific Islander	Hispanic	Non-Hispanic Black	Non-Hispanic White	Multiracial
	n=316	n=3,051	n=6,916	n=3,291	n=33,565	n=3,753
	No. (%)	No. (%)	No. (%)	No. (%)	No. (%)	No. (%)
**Predisposing Factors**						
Family Structure	19 (7.2)	111 (3.9)	256 (3.7)	182 (6.2)	820 (3.0)	110 (3.5)
**Enabling Factors**						
Difficulty Covering Basics	13 (2.2)	108 (3.3)	188 (2.9)	141 (4.8)	595 (2.2)	69 (2.7)
Insurance Type	3 (0.5)	114 (3.4)	171 (3.1)	89 (3.4)	420 (1.6)	57 (2.6)
Usual Source of Pediatric Preventive Care	5 (0.9)	47 (1.9)	88 (1.5)	33 (0.7)	226 (0.7)	23 (0.7)
Personal Doctor or Nurse for Child	1 (0.3)	35 (1.1)	72 (1.2)	34 (0.9)	162 (0.6)	14 (0.5)
**Need Factors**						
Child's Perceived Health	1 (0.3)	5 (0.1)	18 (0.5)	20 (0.7)	64 (0.2)	5 (0.1)
Number of Conditions[a]	37 (13.4)	268 (10.9)	668 (10.1)	396 (11.4)	2,343 (7.5)	252 (6.0)
**Delayed/Missed Pediatric Preventive Care**	20 (8.2)	152 (5.1)	305 (4.5)	216 (7.4)	964 (3.6)	133 (5.1)

[a]Includes the following conditions: allergies, arthritis, asthma, autism (e.g., autism spectrum disorder, Asperger syndrome, or pervasive developmental disorder), attention deficit disorder or attention deficit hyperactivity disorder, anxiety, blindness, blood disorders (e.g., sickle cell disease, thalassemia, or hemophilia), behavioral or conduct problem, concussion or brain injury, cystic fibrosis, cerebral palsy, Down syndrome, diabetes, depression, deafness, developmental delay, epilepsy, frequent or severe headaches including migraines, heart condition, intellectual disability, learning disability, speech or other language disorder, Tourette syndrome, and other genetic or inherited conditions.

*Only variables under study with missing data are included in this Table.

© 2023 Tabet M et al. *JAMA Network Open*.
