# Supplementary material for: Racial and Ethnic Differences in Factors Associated With Delayed or Missed Pediatric Preventive Care in the US Due to the COVID-19 Pandemic
Source: JAMA Netw Open. 2023 Jul 10;6(7):e2322588. doi: 10.1001/jamanetworkopen.2023.22588 (PMC10334219; doi:10.1001/jamanetworkopen.2023.22588)
Supplement: Supplement 2. — Data Sharing Statement [file jamanetwopen-e2322588-s002.pdf]

Data Sharing Statement

Tabet. Racial and Ethnic Differences in Factors Associated With Delayed or Missed Pediatric Preventive Care in the US Due to the COVID-19 Pandemic. *JAMA Netw Open*. Published July 10, 2023. doi:10.1001/jamanetworkopen.2023.22588

Data
Data available: Yes
Data types: Deidentified participant data
How to access data: https://www.census.gov/programs-surveys/nsch/data/datasets.html
When available: beginning date: 12-01-2022

Supporting Documents
Document types: None

Additional Information
Who can access the data: anyone requesting the data
Types of analyses: for any purpose
Mechanisms of data availability: data is freely available online
